# Jakyakgamcho-tang in the relief of delayed-onset muscle soreness in healthy adults: study protocol for a randomized, double-blind, placebo-controlled, crossover design clinical trial

**DOI:** 10.1186/s13063-020-4119-4

**Published:** 2020-02-21

**Authors:** Kyungsun Han, Ojin Kwon, So-Young Jung, In-hwa Park, Man-Suk Hwang, Sun-Young Park, Eui-Hyoung Hwang, Jun-Hwan Lee

**Affiliations:** 1 Clinical Medicine Division, Korea Institute of Oriental Medicine, 1672 Yuseong-daro, Yuseong-gu, Daejeon, 34054 Republic of Korea; 2 Department of Rehabilitation Medicine of Korean Medicine, Spine and Joint Center, Pusan National University Korean Medicine Hospital, Yangsan, 50612 Republic of Korea; 3 Division of Clinical Medicine, School of Korean Medicine, Pusan National University, Yangsan, 50612 Republic of Korea; 4 Korean Medicine Life Science, University of Science & Technology (UST), Campus of Korea Institute of Oriental Medicine, Daejeon, 34113 Republic of Korea

**Keywords:** Delayed-onset muscle soreness, Herbal medicine, Jakyakgamcho-tang, Peony licorice decoction, Shakuyakukanzoto, Shaoyaogancao-tang

## Abstract

**Background:**

Muscle soreness after exercise, called delayed-onset muscle soreness (DOMS), may cause significant changes in muscle function and may increase the risk of sports injuries. Therefore, various therapeutic strategies have been studied to help recovery after exercise. Jakyakgamcho-tang (JGT) is a widely prescribed herbal medicine to treat muscle pain and cramps in traditional Eastern medicine. The aim of this study is to evaluate the effect of JGT for reducing pain and improving muscle damage after exercise.

**Methods:**

This study is a randomized, double-blind, placebo-controlled, crossover design clinical trial. A total of 30 healthy male adults will be recruited. Subjects who voluntarily wish to participate in this study will be hospitalized for 4 days. On the first day, the subjects will perform a standardized treadmill exercise for 1 h to induce DOMS. After the exercise, the subjects will take either JGT or a placebo for 3 days. After a more than 1 week wash-out period, the subjects will repeat the same process with the other drug. Pain intensity, calf circumference, and pain threshold will be measured as outcome measures. Blood tests and blood pressure will be measured as safety assessments. In addition, blood tests for muscle damage and inflammation markers, such as creatine kinase, interleukin-6, and C-reactive protein, will be analyzed.

**Discussion:**

This will be the first trial to assess the effect of JGT on exercise-induced muscle soreness. Our findings will provide valuable data to determine the clinical effects of JGT on DOMS.

**Trial registration:**

Clinical Research Information Sevice, KCT0003457. Registered on 29 January 2019.

## Background

Muscle soreness after exercise, known as delayed-onset muscle soreness (DOMS), occurs after long-term physical activity or unusual exercise [1, 2]. DOMS symptoms, such as pain, discomfort, muscle tenderness, or loss of range of motion, usually occur 12–48 h after exercise and mend within 7 days. DOMS symptoms occur when structural damage to the muscle fibers initiates an inflammatory reaction cascade, which consequently increases inflammatory markers and intramuscular enzymes, such as creatine kinase (CK) [2, 3]. However, the mechanism of DOMS after exercise has not been fully elucidated. Although symptoms disappear over time, eccentric activity may cause significant changes to muscle function and may increase the risk of sports injuries [2]. Analgesics can be used for severe pain, but some studies have demonstrated a lack of efficacy of non-steroidal anti-inflammatory drug treatments [4, 5]. Many studies have attempted to identify ways to alleviate DOMS symptoms, such as massage, cryotherapy, cold-water immersion, and laser acupuncture [6–11]. Various supplements with anti-inflammatory and antioxidant effects have been studied to help recovery after exercise [12–14]. Moreover, there has been increasing interest in herbal medicines to treat DOMS because of their holistic approach and minimal side effects [15].

Jakyakgamcho-tang (JGT), also known as Shakuyakukanzoto, Shaoyaogancao-tang, or Peony licorice decoction, is an herbal medicine widely used to treat acute pain in both skeletal and smooth muscle [16, 17]. JGT is widely prescribed to treat muscle pain and cramps in traditional Korean and Japanese medicine [18, 19]. JGT consists of the herbs *Glycyrrhiza uralensis* Fischer (Glycyrrhizae Radix et Rhizoma) and *Paeonia lactiflora* Pallas (Paeoniae Radix), which contain glycyrrhetic acid, glycycoumarin, isoliquiritigenin, paeoniflorin, and albiflorin as active components [20]. Glycyrrhetic acid inhibits calcium-activated potassium channels, which consequently relax skeletal muscle, while paeoniflorin relaxes muscle fibers by regulating calcium movement near the neuromuscular junction [21, 22]. Another study reported that JGT reduces excess potassium ions from the external space of myofibers, thus reducing muscle pain [23]. Although *Glycyrrhiza uralensis* Fischer and *Paeonia lactiflora* Pallas have known analgesic and muscle relaxation effects, respectively, studies have shown that there is a synergetic effect when both herbs are used together [24–26]. The analgesic effects of JGT have been reported in various animal studies [27, 28]. However, no clinical trials have evaluated the therapeutic effects of JGT on exercise-induced muscle pain.

The aim of this study is to evaluate the effect of JGT on pain reduction and improvements in muscle damage after exercise. The safety and effectiveness of JGT for DOMS symptoms will be demonstrated in a randomized, double-blind, placebo-controlled, crossover design clinical trial. After inducing DOMS with 1-h eccentric exercise, pain intensity, pain threshold in calf muscles, and calf circumference will be measured from 0 to 72 h after exercise along with blood tests for the safety analysis. Additionally, shifts in serum and urinary metabolites will be analyzed using a metabolomics-based analysis to provide insight into the pharmacological mechanism of JGT.

## Methods and design

### Objective

The aim of this study is to assess the therapeutic effect of JGT in the relief of DOMS symptoms. The primary outcome is the difference between the JGT and placebo groups in pain intensity, measured every 12 h from 0 to 72 h after exercise. Our secondary outcomes include the pain threshold in the calf muscles, calf circumference, and serum markers of muscle damage. Furthermore, exercise-induced shifts in serum and urinary metabolites will be analyzed using a metabolomics-based analysis.

### Study design

**Fig. 1:**
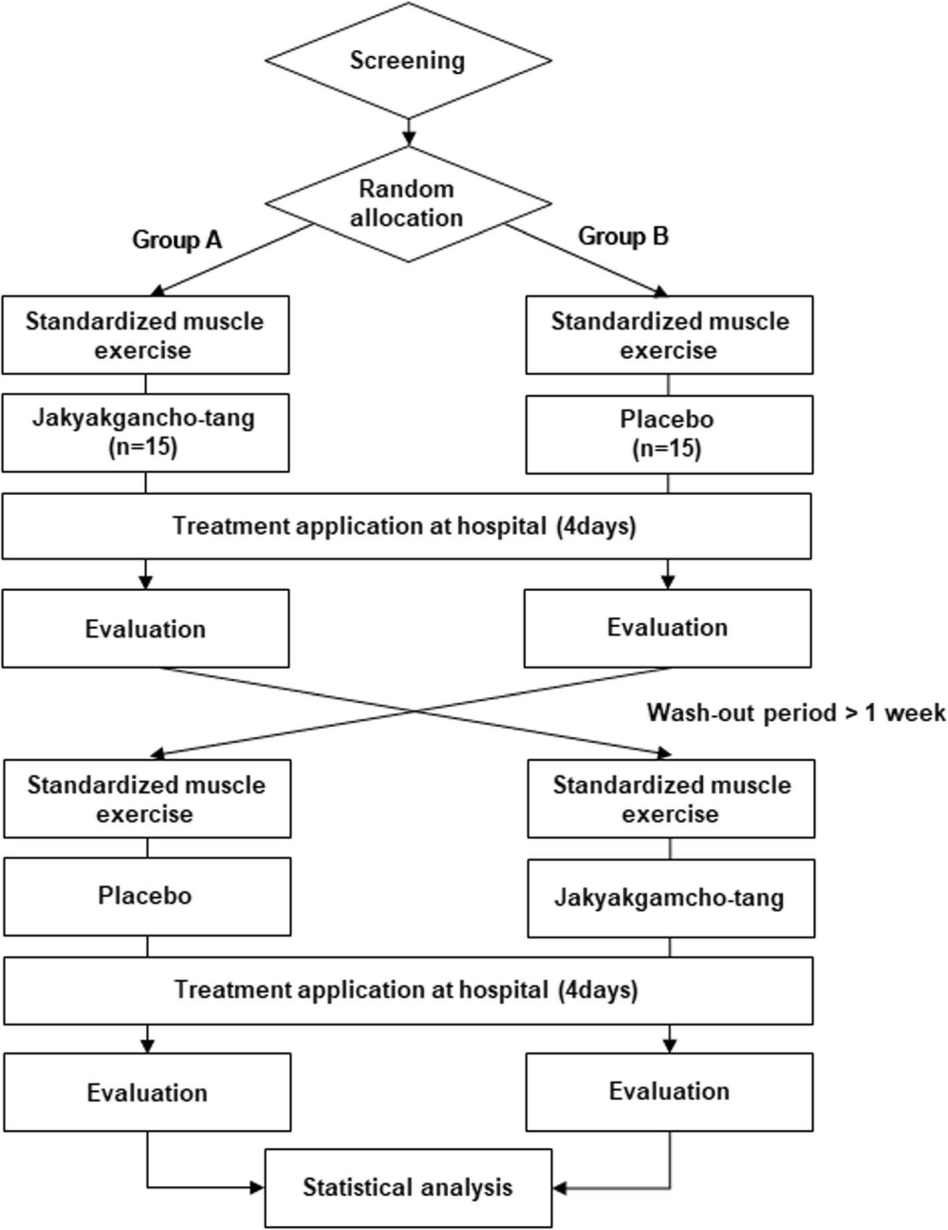
Flow chart of the study

**Fig. 2:**
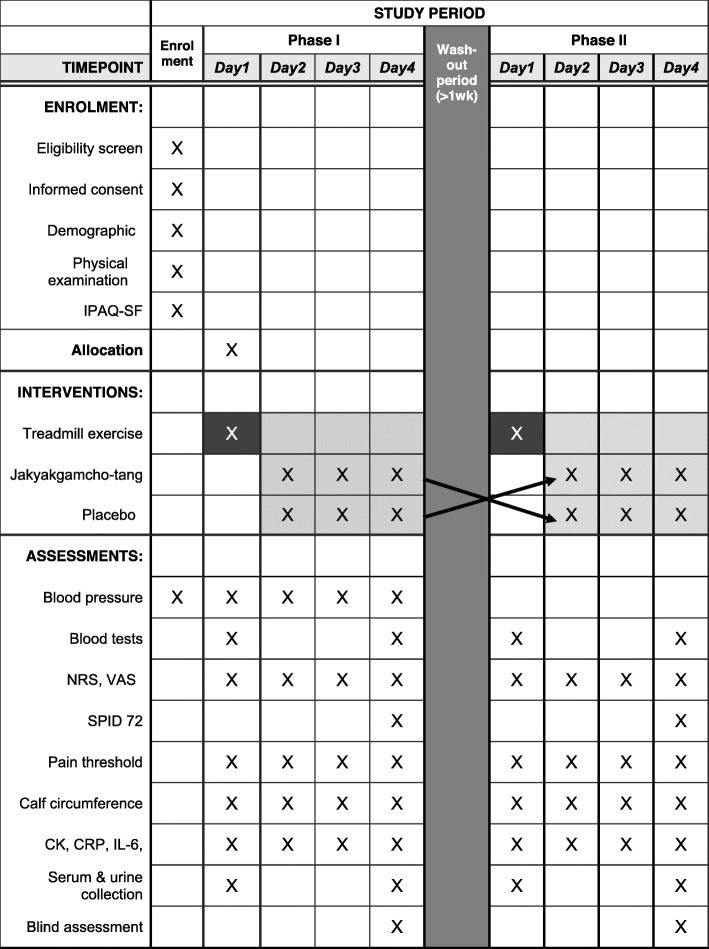
Schedule of enrollment, interventions, and assessments, according to the SPIRIT 2013 statement

This study will be a randomized, placebo-controlled, crossover design, investigator-initiated clinical trial. The trial will be conducted at Pusan National University Korean Medicine Hospital. Thirty healthy male adults will be recruited via hospital bulletin boards and local advertisements. Subjects who voluntarily wish to participate in this study and provide written informed consent will undergo a screening assessment to determine their eligibility. Subjects will be hospitalized for 4 days. On the first day, the subjects will perform eccentric exercise for 1 h to induce DOMS. After exercise, the subjects will be administered either JGT or placebo for 3 days. After a more than 1 week wash-out period, the subjects will repeat the same process with the other drug. The duration of the wash-out period was calculated based on the major component of JGT with the longest half-life. As glycycoumarin has the longest half-life (*T*_1/2_) of 14.9 h [20], we decided that the wash-out period should be 6.21 days or more, which is 10 times the half-life of glycoumarin needed to minimize the carry-over effect of JGT. The overall study flow is shown in Fig. 1. A detailed schedule is given in Fig. 2 and Table 1.

**Table 1:** Time schedule for the in-hospital clinical trial

Day	Day 1				Day 2				Day 3			Day 4			
Time points	15:00	17:00	18:00	19:00	00:00	6:00	12:00	18:00	6:00	12:00	18:00	6:00	12:00	18:00	19:00
Time after exercise		–1 h	0 h	1 h	6 h	12 h	18 h	24 h	36 h	42 h	48 h	60 h	66 h	72 h	
Blood pressure		○		○		○		○	○		○	○		○	
Check for adverse events				○		○		○	○		○	○		○	
Physical examination	○														
Body composition analysis	◎														
IPAQ-SF questionnaire	◎														
Treadmill exercise			○												
Medication				○		○	○	○	○	○	○	○	○		
Randomization			○												
Blood tests		○												○	
Serum CK analysis		○				○		○			○	○		○	
Serum CRP, IL-6		○	○	○	○	○		○							
Pain intensity assessments		○	○	○	○	○		○	○		○	○		○	
Calf circumferences		○	○	○	○	○		○	○		○	○		○	
Pain threshold		○	○	○	○	○		○	○		○	○		○	
Serum & urine collection		○		○										○	
Blind assessment															○

◎ Assess only at the first hospitalization (Phase I)

*IPAQ-SF* International Physical Activity Questionnaire-Short Form, *CK* creatine kinase, *CRP* C-reactive protein, *IL-6* interleukin-6

### Participants

#### Inclusion criteria

Subjects will be considered for inclusion who:

1. Have experienced muscle soreness with a numeric rating scale (NRS) score> 5 within 24 to 30 h after exercise
2. Are healthy male subjects from 19 to 35 years of age without any recognized disease
3. Have a body mass index (BMI) > 18.5 kg/m^2^ and < 30 kg/m^2^
4. Have not exercised regularly for 2 consecutive weeks or more in the last 6 months or more
5. Have voluntarily signed the written consent approved by the institutional review board (IRB) after sufficient explanation prior to the study.

#### Exclusion criteria

Subjects will be excluded if they:

1. Have had an open wound or inflammatory disease within the last 6 months
2. Have neurological or muscular disorders that may affect muscle strength
3. Are missing limbs
4. Have a seriously unstable medical condition determined from a physical examination, such as cardiovascular disease, respiratory disease, gastrointestinal disease, hepatobiliary disease, metabolic disease, endocrine disease, renal disease, urinary disease, or problems with the nervous system or mental health
5. Are taking steroids, analgesics, muscle relaxants, or other medications that the researchers decide to be inappropriate, such as antispasmodics, antidepressants, antidiarrheals, antibiotics, or thrombolytics
6. Have a history of alcohol abuse or drug abuse within the past year
7. Have taken other clinical trial drugs for less than 3 months
8. Have genetic problems, such as galactose intolerance, Lapp lactase deficiency, or glucose-galactose malabsorption
9. Are unwilling or unable to follow the study guidelines
10. Are determined by the researcher to be inappropriate for this study.

### Randomization and allocation concealment

Subjects will be randomly allocated to either the JGT or the placebo group with equal probability. An independent statistician will use SAS version 9.4 software (SAS Institute, Cary, NC, USA) to generate a random allocation list and will send the list to the pharmaceutical company for packing. The random allocation list will assign 30 subjects to group A or B. According to the crossover design, both groups will take JGT and the placebo drug. Subjects in group A will take JGT and those in group B will take the placebo drug during their first hospitalization.

### Blinding

The statistician will send the randomization code to the pharmaceutical company for packing to maintain blindness in both the assessors and the subjects. JGT and the placebo drug will be manufactured by the same pharmaceutical company. The placebo drug will be manufactured in a form similar to that of the JGT. It was confirmed in advance that the taste and flavor will be similar. According to the random sequence, the drugs will be packed in the same form and will be delivered to the hospital. The pharmacist will supply the drug sequentially during the trial period, according to the randomization code. The blinding will be maintained until all 30 subjects have completed the study and the database is locked. To evaluate whether the blinding was successful, a quick questionnaire will be conducted at the end of each hospitalization, asking “Which test drug do you think you were allocated to, JGT or placebo drug? And why do you think so?”

### Exercise protocol for inducing muscle soreness

A standardized treadmill exercise protocol will be performed on the first day of hospitalization to induce DOMS. Previous studies have shown that a downhill running protocol induces stress in the gastrocnemius muscles, as it is an unfamiliar eccentric exercise [29–31]. The subjects will step backwards on a moving treadmill inclined at 13°. Starting with the right leg, the subjects will continue the exercise for 1 h at a speed of 2.2 km/h. The subjects will carry a 5–10 kg weight belt to ensure adequate stimulation to the muscles.

### Intervention

After the treadmill exercise, the subjects will be administered either JGT or the placebo drug three times daily before meals for 3 days (nine times in total). Both the JGT and placebo drug will be manufactured by Kyungjin Pharmaceutical Co., Ltd. (Gyeonggi-do, Republic of Korea) according to Good Manufacturing Practice guidelines. Both drugs will be prepared in the form of light brown granules. The constituents of each drug are shown in Table 2. After the first hospitalization, subjects will be readmitted to the hospital after the wash-out period of more than 1 week. During the second hospitalization, subjects will be administered the other drug, not the one they have taken before, in accordance with the crossover design. No over-the-counter drugs or prescription-based medicines will be permitted throughout the clinical trial. If a subject already has taken a medication during the trial or the subject is suffering from a medical condition that requires medication, the subject will be dropped from the trial.

**Table 2:** Constituents of Jakyakgamcho-tang and the placebo control drug

Test drug	Constituents	Contents	Purpose of use
Jackyakgamcho-tang (JGT)		The amount contained in a single dose (3.0 g)	
	*Paeonia lactiflora* Pallas	2 g	Main components (used as a solid extract 480 mg)
	*Glycyrrhiza uralensis* Fischer	2 g	
	Lactose	1650 mg	Excipient
	Cornstarch	720 mg	Excipient
	Colloidal silicon dioxide	150 mg	Excipient
Placebo		The amount contained in a single dose (3.0 g)	
	Lactose	1800 mg	Excipient
	Cornstarch	785 mg	Excipient
	Colloidal silicon dioxide	160 mg	Excipient
	Caramel color	255 mg	Coloring

### Outcome measures

#### Pain intensity

The primary outcome will be the visual analog scale (VAS) score to measure pain with motion. To assess the perception of DOMS after the treadmill exercise, four separate baseline pain intensity measures will be recorded: the NRS score, a VAS with motion, a VAS at rest, and a VAS after walking downstairs. Pain intensity will be measured before the exercise, immediately after the exercise, and 1, 6, 12, 24, 36, 48, 60, and 72 h after the exercise.

The VAS is one of the most widely used measures of pain intensity, determined on a 100-mm horizontal line, where 0 indicates “no pain” and 100 indicates “the worst imaginable pain” [32, 33]. Assessors will record the length of the point marked by the subject. For the NRS pain score, the subjects will be asked to report overall pain intensity on a single 11-point numeric scale, with 0 indicating “no pain” and 10 representing “the worst imaginable pain” [33]. Although studies have shown that both the VAS and NRS have similar sensitivity in pain assessments, the VAS is believed to have a higher degree of precision [34, 35]. However, the NRS has the advantage that it can be used effectively across all types of disorders because it is easy and simple. To take advantage of each method, the VAS will be used to evaluate the detailed pain intensity with motion, whereas the NRS will be used to evaluate the overall pain intensity.

To assess time-weighted overall pain intensity, the sum of the pain intensity differences (SPID), which is an outcome measure that summarizes the treatment response over a period of time, will be calculated using the NRS scores that were evaluated before exercise and at 24, 48, and 72 h after exercise [36]. The SPID_72_ value will be obtained according to the following calculation: (1) PID (pain intensity difference) = NRS_t_ – NRS_baseline_; (2) SPID_t_ = ∑PID_t_ * (time in hours elapsed since the previous observation). A higher SPID_72_ value indicates larger differences in pain intensity over the 72 h after exercise.

#### Calf circumference

The circumference around both legs will be measured at the level of acupuncture point BL57, horizontally on the ground to obtain a constant reference point. BL57 is in the middle of the calf between the two heads of the gastrocnemius muscle. After marking the acupuncture point with a pen for repeatability, the same measurer will use the same tape to repeat the measurements. The average value will be calculated after measuring three times consecutively. Calf circumferences will be measured before the exercise, immediately after the exercise, and 1, 6, 12, 24, 36, 48, 60, and 72 h after the exercise.

#### Pain threshold

Pain threshold will be measured using a digital algometer (model FPX25, Wagner Instruments, Greenwich, CT, USA). The point of measurement will be acupuncture point BL57 in both calves, at which the rubber tip will be pressed perpendicularly into the muscle with a consistently increasing force of 0.25 kg/cm^2^/s [37]. The values will be obtained when the subject indicates the onset of pain. Each leg will be measured three times after brief resetting times. Average values will be obtained for further analysis. The pain threshold will be measured immediately after measuring the calf circumference.

#### Muscle damage and inflammation

Venous blood samples will be collected to assess muscle damage and inflammation. Plasma CK activity will be used to assess muscle damage. C-reactive protein (CRP) and interleukin-6 (IL-6) concentrations will be measured to evaluate the inflammation induced by eccentric exercise. Whole blood samples will be obtained and centrifuged to isolate the serum. The samples will be stored at − 80 °C and analyzed at the end of the study. As plasma concentrations of the markers will differ depending on the reaction time, each marker will be collected at the appropriate time [38, 39]. Blood samples for CK activity will be collected at six different time points: before exercise, and 12, 24, 48, 60, and 72 h after the exercise. Blood samples for the inflammation markers will be collected at six different time points, including before the exercise, immediately after the exercise, and 1, 6, 12, and 24 h after the exercise.

#### Safety assessment

Blood pressure and heart rate will be measured every day using an automated device. In addition, blood tests will be conducted before and after the drug intervention during each hospitalization. The blood tests will include aspartate aminotransferase, alanine aminotransferase, alkaline phosphatase, total bilirubin, gamma-glutamyl transpeptidase, protein, albumin, blood urea nitrogen, creatinine, red blood cells, hematocrit, hemoglobin, white blood cells, and lipids.

### Exploratory outcomes

#### Metabolomics analysis

During the hospitalization, plasma and urine samples will be obtained three times (before the exercise, 1 h after the exercise, and 72 h after the exercise) to examine the drug-induced and exercise-induced changes in metabolites. Urine samples and plasma will be aliquoted into empty vials and maintained at − 80 °C until further analysis. When the trial has been completed, all samples will be analyzed simultaneously. Non-targeted analysis by mass spectrometry will be used to determine the metabolic changes after exercise and the drug intervention; this evaluation is called “sportomics” [11–13]. Briefly, the supernatants will be obtained by adding acetonitrile or methanol, and the solution will be injected into an ultra-performance liquid chromatography/quadrupole time-of-flight mass spectrometry (UPLC-Q/TOF-MS, Synapt G2Si; Waters, Milford, MA, USA) system. Information from the mass data, including retention time, ion intensity, and m/z will be extracted. After alignment and normalization, the multivariate data matrix will be exported into SIMCA-P (Umetrics, Umea, Sweden) for a multivariate statistical analysis. The ions contributing to separation of the JGT and placebo groups will be further investigated by searching the METLIN and human metabolomics databases.

#### Other outcomes

The degree of pain induced by eccentric exercise can vary depending on the usual momentum and body composition. To determine the effects of usual physical activity and body composition on DOMS symptoms, the Korean version of the International Physical Activity Questionnaire-Short Form (IPAQ-SF) and a body composition analysis will be performed on the first day of hospitalization. The IPAQ-SF consists of seven questions asking about the amount of time spent per week on activities (walking as well as moderate and vigorous activities) and sitting [11, 12]. The total weekly physical activity for each activity category will be estimated by a metabolic equivalent energy expenditure [11]. Body composition parameters, such as body fat percentage, fat mass, and muscle mass will be obtained with the InBody 720 instrument (Biospace, Seoul, Republic of Korea).

### Sample size

As no clinical trial has evaluated the efficacy of JGT on DOMS, a sample size calculation was impossible. A study estimating the sample size for a pilot study proposed 15 subjects per treatment arm as an optimal sample size for the standardized effect size with 90% power and two-sided 5% significance [8].

### Statistical analysis

The statistical analysis will be conducted by an independent statistician using SAS version 9.4 (SAS Institute). The significance level will be set at 0.05 in a two-tailed test. All continuous data will be presented as means and 95% confidence intervals (CIs). Categorical data will be reported as frequencies and percentages. The intention-to-treat (ITT) principle will be applied for the primary analysis. The ITT population will include subjects who meet the full analysis set (FAS) criteria, including subjects who had the primary outcomes assessed once or more except for the baseline measures and who took the test drug at least once. Subjects who do not meet the eligibility criteria, those who did not take the test drug throughout the study, and those who have never been evaluated after the screening visit will be excluded from the FAS dataset. The per-protocol set will also be analyzed, which will only include subjects who have completed the study as a supplementary analysis.

The baseline characteristics of the study subjects will be presented using a descriptive analysis for each group. The primary outcome will be the differences between the VAS score with motion measured at various intervals from 0 to 72 h after exercise. A mixed-effect model for repeated measures (MMRM) will be used to assess differences along with the time effect. The dependent variable will be the change in the VAS score (pain with motion) measured from 0 to 72 h after exercise. The model will include treatment and protocol-specified visits as fixed effects, and subjects as a random factor. Dunnett’s test will be used for multiple comparisons. The results of the secondary effective analysis will be analyzed using the same method as for the primary outcome, in the case of continuous variables with time variation. Additionally, Student’s *t* test or Wilcoxon’s signed-rank test will be used to compare results before and after the treatment within each group. Categorical data will be analyzed using the chi-squared test or Fisher’s exact test. Missing values by maximum likelihood will be considered when the MMRM is used. The last observation carried forward method will be adopted for missing values for Student’s paired *t* test, the Wilcoxon signed-rank test, or repeated measures analysis of variance.

The incidence of serious adverse events (SAEs) and adverse events (AEs) related to the treatment will be analyzed for the safety assessment using the independent *t* test or Wilcoxon’s rank-sum test. The percentage of subjects who experienced one or more side effects during the study will be analyzed using Pearson’s chi-squared test or Fisher’s exact test.

### Data management and monitoring strategies

All source documents, including informed consent forms, questionnaires, and worksheets, will be collected in compliance with standard operating procedures. The data will be collected on an electronic data capture system through electronic case report forms (eCRFs) using the Medidata RAVE data management system (Medidata Solutions Inc., New York, NY, USA). The Korea Institute of Oriental Medicine will be responsible for quality control throughout the study. An independent clinical research associate will regularly monitor the overall process to determine if the trial is performed in accordance with the protocol.

### Adverse events

During the hospitalization, subjects will be asked if they have any physical discomfort other than DOMS symptoms. Occurrence of any AEs will be recorded on the eCRF after assessing severity and causality. If a SAE occurs, it will be reported to the IRB as soon as possible. If suspected unexpected serious adverse reactions occur, they will also be reported to the Ministry of Food and Drug Safety according to the relevant regulations.

## Discussion

This is a protocol for a randomized, double-blind, placebo-controlled, crossover-designed clinical trial to study the efficacy and safety of the herbal medicine JGT in the relief of DOMS in healthy male adults. The strength of this study lies in the rigorous design. In general, DOMS is difficult to test in a clinical trial because of the short duration of the symptoms. A standardized method for pain induction and a rigorously designed clinical trial will provide reliable results on the therapeutic effects of JGT in DOMS. The Standard Protocol Items: Recommendations for Interventional Trials (SPIRIT) checklist is attached as Additional file 1. This study will provide a better understanding of the mechanisms of JGT for relieving muscle pain in general, as DOMS is an important experimental model to evaluate analgesic efficacy and to study movement-related pain [40, 41]. We expect to provide novel insights into potential biological mechanisms of JGT through a metabolomics approach. In this protocol, the inclusion criteria of the study participants will be narrowed to male adults, and participants will be hospitalized during the intervention. Therefore, the results of the metabolomics analysis are expected to yield reliable results, because factors affecting metabolites, such as age, gender, and food intake, will be controlled [42, 43]. Moreover, as this is a 2 × 2 crossover design study, each subject will serve as his own control to reduce individual variations.

To our knowledge, no clinical trial has evaluated the therapeutic effects of JGT on exercise-induced muscle pain. Several clinical trials have evaluated the antispasmodic and analgesic effects of JGT. Clinical trials of JGT to reduce pain have mostly considered pain caused by complications, such as muscular cramps of patients on hemodialysis and myalgia caused by chemotherapy [44–48]. Although it is widely prescribed to manage muscle soreness and myalgia, it is difficult to find any evidence other than a clinical study about JGT on painful muscle cramps in patients with lumbar spinal stenosis [49]. A retrospective case series study with 37 patients treated with JGT for muscle spasms and pain was published in 2004 [16]. However, the current clinical trial will be the first to assess the effect of JGT on exercise-induced muscle soreness. This study will provide valuable data to determine the clinical effects of JGT on exercise-induced pain.

### Trial status

This study was approved by the Pusan National University Korean Medicine Hospital IRB (IRB approval number 2018013). The most recent version of the protocol is version 1.4 (3 January 2019), and this was also approved by the IRB. Recruitment began on 7 February 2019. This study is expected to be completed by August 2019.

## Supplementary information

**Additional file 1.** SPIRIT 2013 Checklist.

## Data Availability

The final dataset of the clinical trial will be accessible to all authors and the Ministry of Food and Drug Safety (MFDS).

## Abbreviations

AE: Adverse event
CK: Creatine kinase
CRP: C-reactive protein
DOMS: Delayed-onset muscle soreness
eCRF: Electronic case report form
FAS: Full analysis set
IL-6: Interleukin-6
IPAQ-SF: International Physical Activity Questionnaire-Short Form
IRB: Institutional review board
ITT: Intention-to-treat
JGT: Jakyakgamcho-tang
MET: Metabolic equivalent
MMRM: Mixed-effect model for repeated measures
NRS: Numeric rating scale
PID: Pain intensity difference
SAE: Serious adverse event
SPID: Sum of pain intensity differences
VAS: Visual analog scale
